# All-dielectric structural coloration empowered by bound states in the continuum

**DOI:** 10.1515/nanoph-2024-0367

**Published:** 2024-10-07

**Authors:** Hong Zheng, Haiyang Hu, Thomas Weber, Juan Wang, Lin Nan, Bingsuo Zou, Stefan A. Maier, Andreas Tittl

**Affiliations:** Beijing Key Laboratory of Nanophotonics and Ultrafine Optoelectronic Systems, School of Physics, Beijing Institute of Technology, Beijing 100081, China; Chair in Hybrid Nanosystems, Nanoinstitute Munich, Faculty of Physics, Ludwig-Maximilians-Universität München, 80539 München, Germany; Guangxi Key Lab of Processing for Nonferrous Metals and Featured Materials, School of Resources, Environments and Materials, Guangxi University, Nanning 530004, China; School of Physics and Astronomy, Monash University, Clayton, Victoria 3800, Australia; Department of Physics, Imperial College London, London SW72AZ, UK

**Keywords:** color printing, bound states in the continuum, all-dielectric

## Abstract

The technological requirements of low-power and high-fidelity color displays have been instrumental in driving research into advanced coloration technologies. At the forefront of these developments is the implementation of dye-free approaches, which overcome previous constraints related to color resolution and fading. Resonant dielectric nanostructures have emerged as a promising paradigm, showing great potential for high efficiency, high color saturation, wide gamut palette, and image reproduction. However, they still face limitations related to color accuracy, purity, and simultaneous brightness tunability. Here, we demonstrate an all-dielectric metasurface empowered by photonic bound states in the continuum (BICs), which supports sharp resonances throughout the visible spectral range, ideally suited for producing a wide range of structural colors. The metasurface design consists of TiO_2_ ellipses with carefully controlled sizes and geometrical asymmetry, allowing versatile and on-demand variation of the brightness and hue of the output colors, respectively.

## 1 Introduction

Generating vibrant and well-defined colors has been a motivating factor for the advancement of optics since ancient times [1], [2], [3]. Dyes and pigments are the predominant materials utilized for color rendering, selectively absorbing parts of the visible spectrum while reflecting the others, resulting in reduced brightness and a limited gamut [4]. In addition, further developments of this passive coloration approach are restricted by wavelength-limited resolution, color fading over time, and vulnerability to photobleaching upon exposure to intense ultraviolet radiation [5]. To address the above concerns, structural coloration, stemming from the precise manipulation of light–matter interaction through nanostructured materials, has become an alternative approach to tackle the obstacles of dyes and pigments [6], [7]. Different from pigment- or dye-based methodologies, such coloration techniques provide several advantages, including superior chromaticity and color gamut, improved spatial resolution, long-lasting coloration, and manufacturing scalability [8], [9], [10]. Plasmonic resonances of metallic nanostructure have been intensively studied to demonstrate high-resolution color printing beyond the optical diffraction limit, precisely manipulating the geometry of nanostructures to tailor the reflectance and transmittance spectra [11], [12], [13], [14]. However, intrinsic metallic loss in plasmonic systems can impede the generation of sufficiently brilliant coloration in the visible spectrum [8].

Alternatively, high refractive index dielectric nanostructures provide an effective way to overcome these challenges [15], [16], [17], [18], [19], [20], [21], [22], [23]. As of late, all-dielectric metasurfaces have emerged as a promising technology to produce vibrant colors by exploiting the Mie resonances of individual nanostructures. For instance, TiO_2_ nanostructures supporting electric and magnetic dipole resonances have proven to be highly tunable [20] and possess high color saturation [16]. Similarly, Si_3_N_4_ metasurfaces based on Rayleigh anomalies were employed to suppress high-order Mie resonances at relatively short wavelengths and generate vivid colors [22]. Additionally, color saturation has been improved even further by employing multilayer dielectric stacked nanostructures [18] and matching refractive index layers [21]. However, the above-mentioned metasurfaces often lack the ability to easily adjust color brightness. Recently, crystalline silicon metasurfaces have been shown to facilitate tailored hue, saturation, and color intensity modulation [19]. Notwithstanding, the implementation of a spatial multiplexing strategy significantly deteriorates linewidth, and high aspect ratio surface features can pose nanofabrication challenges.

All-dielectric metasurfaces supporting photonic bound states in the continuum (BIC) have gained considerable attention for their potential in nanoscale lasing [24], [25], [26], chiral optics [27], [28], [29], nonlinear photonics [30], [31], [32], biomolecular sensing [33], [34], [35], and photocatalysis [36] due to their exceptional spectral selectivity, strong light confinement, and significant enhancement of electric fields. A true BIC, originally described in quantum mechanics [37], is a mathematical concept with an infinite *Q* factor and vanishing resonance width in an open system, which cannot couple to any external radiation channel [38], [39], [40], [41]. Slight perturbations can break the pure BIC condition and create quasi-BIC (q-BIC) modes, which can be excited and observed from the far field [41], [42], [43]. Symmetry-protected BICs have received growing attention among the various types of BICs, due to their high optical signal contrasts, experimental robustness, and straightforward measurements using brightfield microscope [33], [35], [44], [45]. Specifically, breaking the in-plane inversion symmetry of the BIC unit cell geometry induces energy dissipation from the resonator system through the radiative loss channel, allowing such resonances to be observed from the far field in reflectance and transmittance spectra. The leveraging of q-BIC modes provides an effective approach to constructing the ideal saturated color pixel, sometimes referred to as a Schrödinger’s pixel [46], [47], [48], [49]. Yet the considerable intrinsic losses in amorphous Si towards lower wavelengths have impeded efforts to achieve highly saturated blue and green pixels using q-BIC resonances. Furthermore, symmetry-protected BICs can be fine-tuned by modifying their geometrical asymmetry, resulting in changes in their intensity and enabling tunable brightness, which has so far been overlooked. Besides, achieving color brightness controlling metasurfaces incorporating liquid crystals is hindered by complex device architectures and incomplete spectral response for full-color displays [50].

Here, we demonstrate a TiO_2_ metasurface supporting symmetry-protected BICs, which achieves high reflectance, pure color rendition, a wide color gamut, and straightforward control over the color brightness. The dielectric metasurface, composed of a zig-zag array of elliptical TiO_2_ resonators on a glass substrate, exhibits sharp reflectance spectra with low background, thus providing saturated and bright reflectance spectra in the visible region. Fine-tuning of the geometric asymmetry factor allows precise control over the radiative loss, enabling precise tailoring of the reflectance amplitude (i.e., the color brightness). Significantly, the higher-order modes have been successfully suppressed to ensure better coloration. In simulations, the geometry of the q-BIC unit cell is optimized to realize the brightest and most saturated color via the radiative decay rate. Experimentally, we obtain a wide color gamut (around 130 % of the sRGB gamut), of which more than 80 % are covered with resonances exhibiting a reflectance amplitude *R* > 0.8. As proof-of-concept demonstrations, we further fabricated several color printing metasurfaces.

## 2 Results and discussion

### 2.1 Design of BIC-driven optical metasurfaces

**Figure 1:**
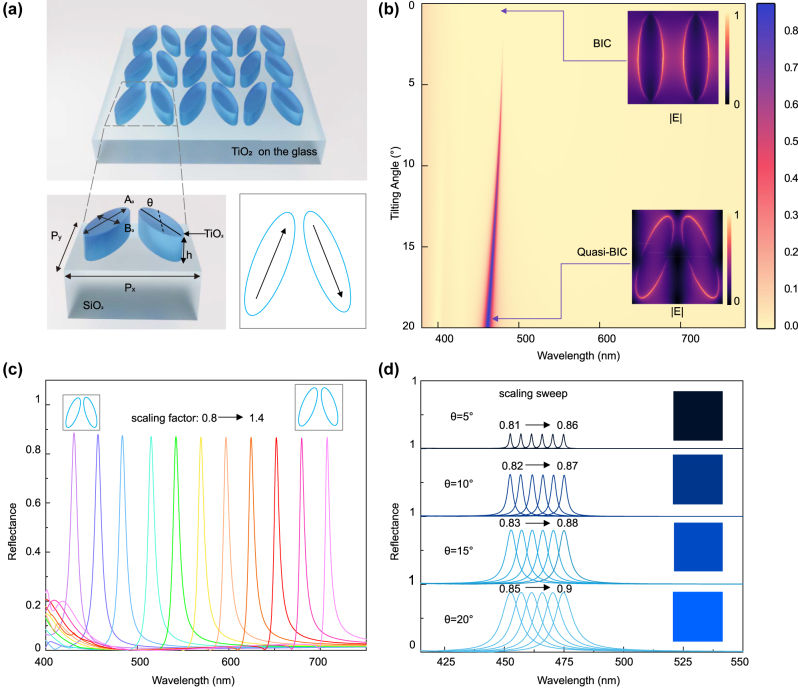
Numerical analysis of symmetry-protected BIC metasurfaces for color response and continuous intensity tunability. (a) Schematic diagram of the TiO_2_ metasurface supporting symmetry-protected BICs together with a sketch of the BIC working principle of opposing dipoles. (b) Color coded map of reflectance spectra as a function of tilting angle and wavelength, while keeping the scaling factor *S* fixed at 0.8. The structure height (*h*) is 120 nm. The inset shows the electric near-field distribution for corresponding BIC and quasi-BIC. (c) Simulated reflectance spectra of BIC resonances covering the entire visible spectrum by adjusting the scaling factor. (d) Simulated reflectance spectra of BICs with different scaling factors *S* and asymmetries *θ*, respectively. The insets show the corresponding calculated colors for the spectra highlighted by the dashed line [52].

Vibrant and brightness-tunable colors are generated in reflection by engineering the radiative decay rate of the BIC-driven resonances, achieved by manipulating the asymmetry of the underlying unit cell geometry of the metasurface. The metasurface is composed of two elliptical TiO_2_ nanostructures on top of a glass substrate, where the main ellipse axes are tilted towards the *y*-axis to produce a zig-zag array (Figure 1a). The chosen two-ellipse BIC unit cell geometry enables precise control of coupling to far-field radiation, resulting in high signal modulation, low spectral background, and fabrication robustness [33], [34], [49], [51]. The major (*A*_0_) and minor (*B*_0_) axes of the ellipses are set to 300 nm and 100 nm, respectively. The pitch along the *x* and *y* direction of the 2D nanostructure array is fixed at 330 nm. To spectrally shift the BIC resonances, a multiplicative scaling factor *S* is applied to these lateral geometrical parameters. The height of the nanostructures is fixed with a value of 120 nm. The asymmetry parameter is defined by the tilting angle *θ* between the *y*-axis and the long axes of the ellipses. The q-BIC metasurfaces are composed of amorphous TiO_2_, with *n* and *k* values as shown in Figure S1. The optical constants of TiO_2_ were obtained from spectral ellipsometry.

To evaluate the color performance of the BIC-driven metasurface, we first numerically investigated the reflectance spectra under incident *x*-polarized light using a finite-element frequency-domain Maxwell solver (for details see Section 4). Under normal incident *x*-polarized excitation, we observe sharp resonances with a low spectral resonance background, where the resonance linewidth is controlled by the asymmetry parameter introduced by the tilting angle (Figure 1b). Specifically, a symmetry-protected BIC structure (*θ* = 0°) would be inaccessible for electromagnetic waves from the far field. As the symmetry is broken and the structure becomes asymmetric (*θ* > 0), the resonance will broaden due to the increased radiative decay rate. Equations for the far-field reflectance and the relationship between the radiative decay rate and the asymmetry factor are given in Supplementary Note 1. Notably, the metasurface with a tilting angle of 20° delivers a sharp resonance with high intensity and low spectral background, which is ideal for obtaining vibrant colors. Moreover, the geometric parameters were optimized to guarantee a low reflectance baseline over the visible spectrum. In addition, this architecture allows for straightforward resonance tuning via multiplying the unit-cell dimensions with a scaling factor *S* varying from 0.8 to 1.4, as given by *A* = *S*·*A*_0_, *B* = *S*·*B*_0_, *P* = *S*·*P*_*x*_. Linear tuning of the resonance position can cover the entire visible region (Figure 1c). As the scaling factor increases, the reflectance peak position redshifts from 434 to 702 nm. In general, if the resonator volume increases, the resonance background will be higher, or a new higher-order mode will be generated, resulting in reduced color quality. Conversely, decreasing the corresponding parameter values will cause lower resonance modulation, giving rise to dim colors and a narrower gamut. Besides, extending the tilting angle beyond 20° is likely to further decrease color saturation, as shown in the Figure S2.

The resonance properties discussed above directly influence the perceived color of our TiO_2_ metasurfaces. While the resonance linewidth determines its purity, the resonance modulation determines how much light is reflected, giving a measure of its brightness. The modulation of the BIC resonances can be tuned via asymmetry parameter *θ*, which however induces a spectral redshifts of 20 nm when *θ* is changed from 20° to 0°, therefore effectively changing brightness and hue simultaneously. To disentangle these properties, we compensate the shift induced by the degree of asymmetry by rescaling the metasurface unit cell (Figure 1d). A similar analysis for adjusting the brightness of the green and red pixels is shown in Figure S3, highlighting the potential of our all-dielectric metasurface approach for color filtering devices. Moreover, Figure S4 shows the reflectance spectra of blue, green and red color pixels with different tilting angles of the nanostructures varying from 0 to 20° after a scaling sweep. The RGB pixel spectral profiles remained, but with reduced brightness, preserving hue and saturation.

### 2.2 Fabrication and experimental validation for achieving high color saturation

**Figure 2:**
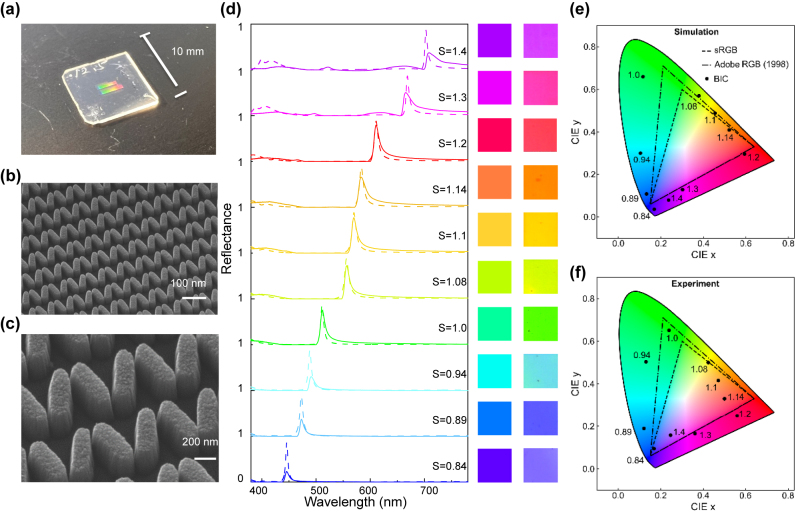
Experimental color performance of the metasurfaces. (a) Photograph of a manufactured large-scale all-dielectric metasurface, employed in subsequent reflectance measurements. (b, c) SEM micrographs of the metasurface confirm the homogeneity of the nanofabrication. (d) The experimental (solid lines) and simulated (dashed lines) spectra depict several rainbow colors observed in distinct samples. The side illustration shows the recorded (right) and calculated (left) structural colors. The chromaticity coordinates on the CIE 1931 diagram were derived from both simulated spectra (e) and measured spectra (f).

The TiO_2_-driven structural coloration metasurface was fabricated via electron-beam lithography on a glass substrate. The fabrication process is depicted in Figure S5 and described in details in the Section 4. Figure 2a shows a full-color photograph of a dielectric chip with an 18 × 18 array of metasurfaces obtained by nanostructuring a 120 nm thick TiO_2_ film on a glass substrate, and where every metapixel has an approximate side length of 60 μm. Scanning electron microscopy (SEM) images of the fabricated metasurfaces demonstrate the accurate and uniform reproduction of each unit cell in the metasurface (Figure 2b). The minimum gap size between resonators is about 12 nm (Figure 2c). While deviating from the numerically predicted optimum values, we selected an asymmetry parameter *θ* of 20°, because it offered enhanced reproducibility and stability within the constraints of our nanofabrication processes. Figure 2d presents the experimentally measured spectra, which closely resemble the simulated results with scaling factor *S* varying from 0.84 to 1.4, where the inset shows the simulated color. Significantly, the metasurface spectrum exhibits sharp peaks at the given design wavelengths while maintaining low resonance background. As the scaling factor increases, the high-reflectance spectral region shifts from 446 to 704 nm. Both the peak positions and the FWHMs match the simulated results well. We attribute the slight discrepancies between the measured and simulated reflectance spectra to fabrication variations. The reflectance peak decreases to a value of 0.35 within the blue region (*S* = 0.84, 0.89, 0.94), which is attributed to the onset of intrinsic absorption in the amorphous TiO_2_ [53]. Improvements in TiO_2_ quality, such as the use of TiO_2_ in its crystalline phase [54], would lead to heightened performance in the blue region and could be realized through processes such as atomic layer deposition [55]. For larger unit cell (*S* = 1.3, and 1.4), we reach the boundary of this regime, subsequently the quasi-BIC modes shift towards the grating modes, which decreases their modulation in experiment. The details of the optical measurements are presented in the Section 4.

To gain a deeper understanding of the metasurface’s color saturation and spectral coverage, we computed the color gamut using color-matching functions as defined by CIE (see Supplementary Note 2). The outcomes are presented in Figure 2e and f. The chromatic coordinates corresponding to the experimentally measured spectra show a reasonable agreement with their simulated counterparts. In the experiment, the color gamut size is approximately 130 % of the sRGB gamut, as well as 96 % of the Adobe RGB space. We note that due to the normalization of the reflectance data (Figure 2d), values below zero can occur. These have been omitted (by setting them to zero) in order to avoid an overestimation of the gamut. A comparison with the raw uncorrected dataset is shown in Figure S6.

### 2.3 Experimental demonstration of color gradients

**Figure 3:**
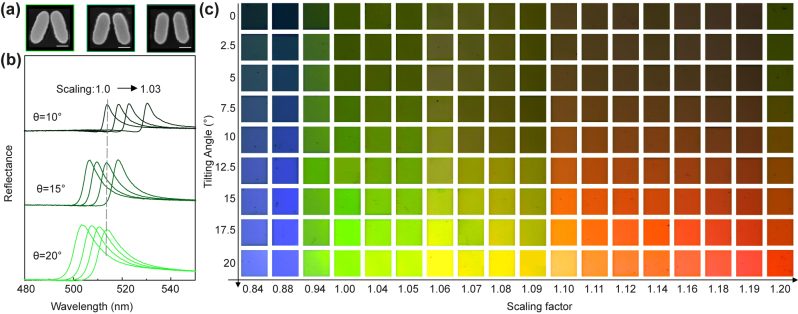
Continuous hue and brightness tuning of q-BIC color pixels. (a) Top-view SEM images show unit cells with varying *θ* from 10° to 20°. Scale bar: 100 μm. (b) Measured reflectance spectra of the green pixels with tilting angle varying from 10 to 20°. (c) Photograph of the color palette with varying scaling factors and tilting angles of the ellipses. Each metapixel is 40 μm × 40 μm.

Color intensity (brightness) along with chromaticity are vital components of the structural coloration response. The intensity of the reflectance amplitude can be modulated by controlling the asymmetry parameter *θ*. To demonstrate this experimentally, we fabricated metasurfaces with asymmetry parameters of *θ* = 20°, 15°, and 10° (see SEM images in Figure 3a). As the asymmetry parameter increases, Figure 3b shows a rise in the reflectance modulation, enabling customization of the color intensity. The reflectance spectra exhibit a noticeable blue shift for increasing asymmetry, in line with the numerical predictions. We further optimize the scaling factor of the BIC metasurface at different tilting angles, with the aim of realizing a continuous modification of the color brightness without changes in hue and saturation. Figure S7 shows the measured spectra of the three primary RGB colors before and after scaling factor adjustment.

Figure 3c displays photographs of 126 samples featuring distinct scaling factors and tilting angles. It can be observed that the hue is gradually varied from blue to red with increases of *S* from 0.84 to 1.2. In addition, a color brightness transition can be realized when changing the tilting angle of the nanoantenna from 0 to 20° in steps of 2.5°. All the results were obtained by photographing the metasurface with an optical microscope (Olympus EP50, see Section 4 for details).

### 2.4 Color image printing with TiO_2_ metasurfaces

**Figure 4:**
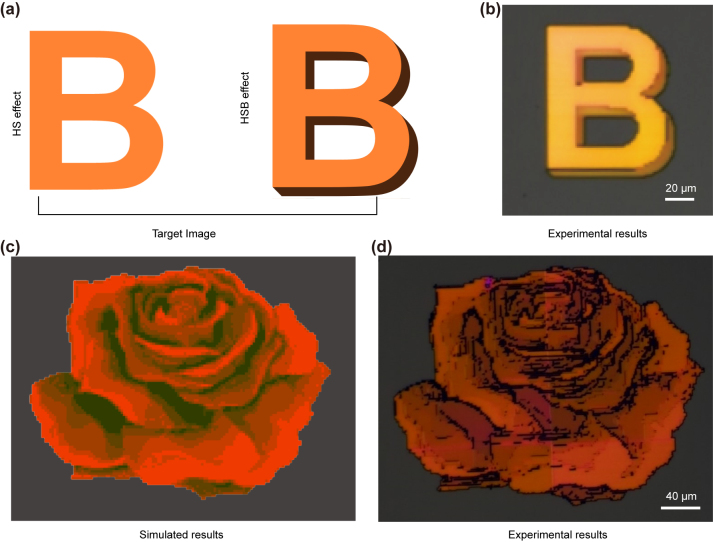
All dielectric nanopainting. (a) In comparison to hue-saturation (HS) images, HSB (hue-saturation-brightness) images provide a more comprehensive representation of light-dark information and (b) optical image based on the proposed metasurface. (c) Simulated and (d) experimental results of the encoded color image.

The manipulation of color brightness results in a shadow-rendering effect. As a proof-of-concept demonstration, a pattern incorporating different color brightnesses was chosen as the target image (Figure 4a). A microscope image of the result is depicted in Figure 4b, which highlights the different levels of brightness caused by nanostructures tilted at different angles. By encoding color hue and brightness information into nanostructures with different scaling factors and tilting angles, the printed image can include a variety of highly saturated colors when illuminated with linearly polarized white light. This characteristic enabled the design of a parameter-matching approach to reproduce colored paintings. As illustrated in Figure S8, the procedure involves pixelating the target image, segmenting it into RGB channels, and subsequently associating the color values of each pixel with the closest colors present within the metasurface’s color palette. In accordance with this methodology, Figure 4c showcases the simulated image. The colored metasurface generates a high-resolution image when illuminated with *x*-polarized white light, showcasing the seamless blending of the darker peripheral sides with the black background, accompanied by smooth brightness transitions (Figure 4d). Moreover, we evaluate the influence of various area sizes on the q-BIC resonance, which is known to deteriorate with smaller array sizes. We find that color pixel sizes ranging from 60 μm to 5 μm maintain bright colors (Figure S9). The array size primarily affects the brightness of the colors instead of their saturation [56]. Additionally, this investigation reveals that even with reduced numbers of unit cells (14 × 14, 16 × 16, and 18 × 18) for the red, green, and blue colors, the pixels retain their ability to deliver highly saturated colors, which opens up the possibility of full-color stereoscopic printing in the future. To visualize structural variations at different location in Figure 4b and d, we have included detailed CAD drawings in Figure S10 to better illustrate these differences.

## 3 Conclusions

We have demonstrated a TiO_2_ metasurface design based on symmetry-protected BICs to realize structural color painting techniques. The q-BIC modes generate sharp reflectance spectra that produce saturated color pixels covering the full visible spectral range. Notably, by varying the asymmetry parameter of the individual metasurface unit cells, we also achieve control over the color brightness. The proposed metasurfaces offer an alternative way to obtain individual control over the hue and brightness of the generated colors. Even though our current design utilizes polarized q-BIC modes, growing attention is directed towards the development of polarization-independent BIC-driven metasurfaces, hinting at future applications in structural coloration [57]. Finally, the unique characteristics of BIC-driven structural color metasurfaces offer a promising avenue for the generation of distinct visual effects, particularly polarization-encrypted anti-counterfeiting applications [58], [59].

## 4 Experiment and methods

### 4.1 Numerical simulations

The numerical simulations of the q-BIC metasurfaces were carried out using the finite element solver contained in CST Microwave Studio (Dassault Systèmes), where periodic boundary conditions were applied and the incident light (*k*) was set to be perpendicular to the metasurface plane with *x*-polarization (TM). Experimentally measured optical constants obtained through ellipsometry were implemented for TiO_2_ to improve the agreement between simulations and experiments. The SiO_2_ substrate was assumed to be lossless with a refractive index of *n* = 1.5.

### 4.2 Nanofabrication

TiO_2_ was sputtered on SiO_2_ coverslips at a pressure of 5 × 10^−7^ Torr at a rate of 0.2 Å/s. Afterward, the positive electron beam resist poly(methyl methacrylate) (PMMA, 950 K, A4, Microresist) was spun onto the sample with a soft-baking steps of 3 min at 180 °C. An electrically conductive polymer (Espacer 300Z) was coated on top of the resist to avoid electron charge accumulation and thus pattern distortions. The lithography pattern was defined using electron beam lithography (Raith eLine plus) with an acceleration voltage of 30 kV, aperture size of 15 μm, a working distance of 10.6 mm, and an area dose of 150 μC/cm^2^. After the conductive polymer was washed off in a water bath for 10 s, the PMMA layer was developed in a 3:1 isopropanol (IPA): methyl isobutyl ketone (MIBK) solution for 135 s with a subsequent 30 s bath in pure IPA. The metal hard mask consisted of a 50 nm chromium (Cr) layer which was deposited via electron beam evaporation. The lift-off process was conducted in Microposit Remover 1165 overnight at 80 °C, followed by reactive ion dry etching in an RCP-RIE system using an SF_6_/Ar plasma. Finally, the chromium layer was removed by wet etching with chromium etchant (Sigma-Aldrich).

### 4.3 Optical measurement

The refractive indices and extinction coefficients of TiO_2_ films were extracted from optical modeling of measured variable-angle spectroscopic ellipsometry data (J.A. Woollam, M2000XI-210). Ellipsometry spectra were acquired over a range of 210–1,690 nm and at four different angles between 65 and 80°.

Reflectance measurements of the fabricated metasurface samples were carried out with a WiTec optical microscope comprising 10× objective (NA = 0.25, Zeiss, Germany) under the illumination of the polarized broadband light source (Olympus TH4-200). The reflectance spectra are normalized to the reflectance response of a silver mirror.

Optical bright-field images were acquired on an Olympus EP50 microscope using a 10× objective (NA = 0.25) and linearly polarized white light from an LED. For the bright-field images, the white balance was calibrated on a Spectralon Diffuse Reflectance Standard (Labsphere).

## Supplementary Material

Supplementary Material Details
