# Supplementary material for: All-dielectric structural coloration empowered by bound states in the continuum
Source: Nanophotonics. 2024 Oct 7;13(23):4327–35. doi: 10.1515/nanoph-2024-0367 (PMC11542552; doi:10.1515/nanoph-2024-0367)
Supplement: Supplementary file 1 — Supplementary Material Details [file j_nanoph-2024-0367_suppl_001.docx]

**Supporting information for**

All-Dielectric Structural Coloration Empowered by Bound States in the Continuum

Hong Zheng^1,2^, Haiyang Hu^2^, Thomas Weber^2^, Juan Wang^2^, Lin Nan^2^, Bingsuo Zou^1,3^, Stefan A. Maier^4,5^, and Andreas Tittl^2*^

1 Beijing Key Laboratory of Nanophotonics and Ultrafine Optoelectronic Systems, School of Physics, Beijing Institute of Technology, Beijing 100081, China

2 Chair in Hybrid Nanosystems, Nanoinstitute Munich, Faculty of Physics, Ludwig-Maximilians-Universität München, 80539 München, Germany

3 Guangxi Key Lab of Processing for Nonferrous Metals and Featured Materials, School of Resources, Environments and Materials, Guangxi University, Nanning 530004, China

4 School of Physics and Astronomy, Monash University, Clayton, Victoria 3800, Australia.

5 Department of Physics, Imperial College London, London SW72AZ, UK.

*Andreas.Tittl@physik.uni-muenchen.de

**Supplementary Note 1**: Determination of reflectance equations

The far-field reflectance can then be calculated according to temporal coupled mode theory (TCMT) approach^1,2^, which are associated with the radiative $\gamma_{rad}$ and intrinsic $\gamma_{int}$losses, respectively. The reflectance R can be expressed as:

$$R={|\frac{\gamma_{rad}}{\gamma_{int}+\gamma_{rad}+i(\omega-\omega_{0})}|}^{2}$$

And the relationship between $\gamma_{rad}$ and asymmetry factor $\alpha$ follows the characteristic quadratic relationship:

$$\gamma_{rad}\sim\alpha^{2}=\sin^{2} \theta$$

**Supplement Note 2: Calculation of chromaticity coordinates**

We evaluate the chromaticity of our structural colors based on the CIE 1931 xyY color space using the python package “colour” using the 2 Degrees Standard Observer color matching functions and the D65 standard illuminant.

Because experimental reflectance spectra were normalized to a bare TiO_2_ film, reflectance values can dip below zero. As this would artificially inflate the color gamut, we clip these values and set them to zero, which yields good agreement with simulations, without underestimating the gamut by shifting the complete reflectance spectra such that no value falls below zero, which would exaggerate the non-resonant part of the spectrum.

***
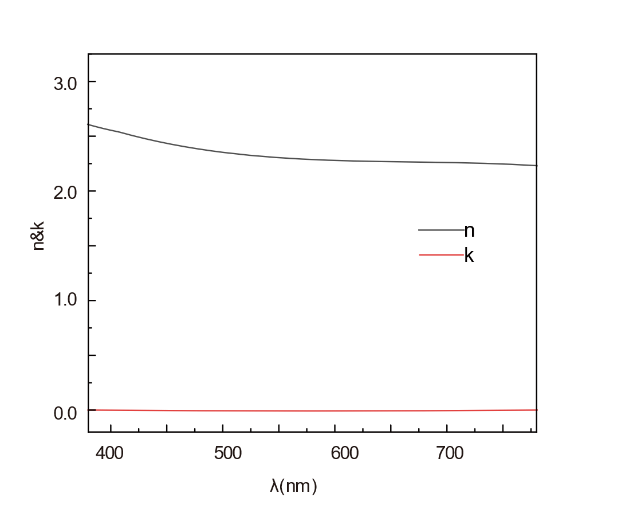
***

Figure S1. The refractive index (n) and extinction coefficient (k) for the amorphous TiO_2_ films. The light extinction coefficient (k) of titanium dioxide (TiO_2_) can be influenced by its quality. Despite the apparent dissimilarity in k values illustrated in Figure S1, it is imperative to acknowledge the utilization of amorphous TiO_2_ in our investigation, which manifests a detrimental impact in the blue spectral range. Nevertheless, it is crucial to underscore that this outcome does not constitute a conceptual constraint inherent to our methodology, as mitigation strategies can be implemented through materials engineering techniques.


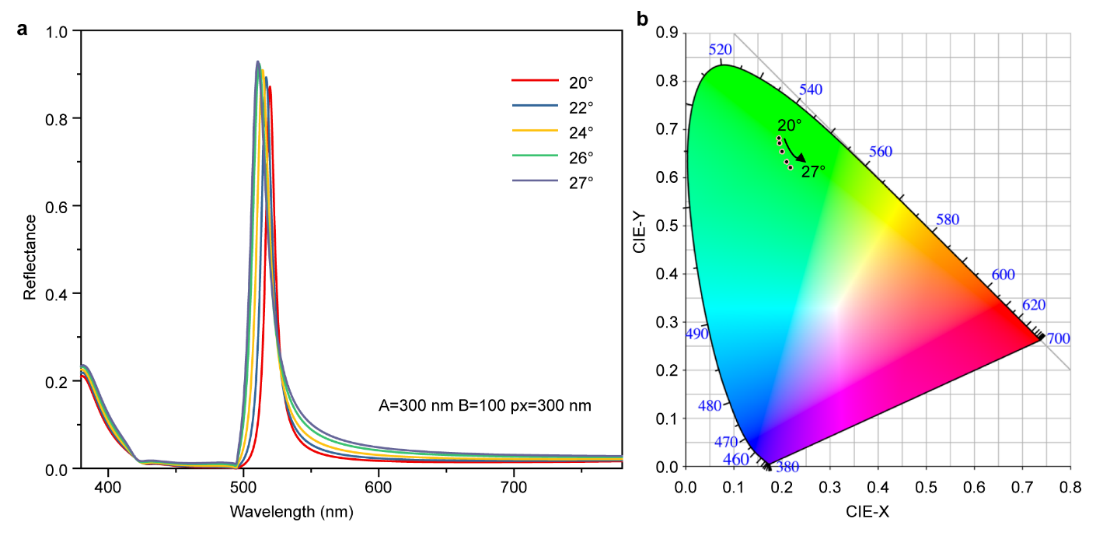


Figure S2. (a) The simulated reflectance spectra versus the titling angle of the nanoresonators. (b) Chromatic coordinates in the CIE 1931 diagram obtained from the simulated spectra.


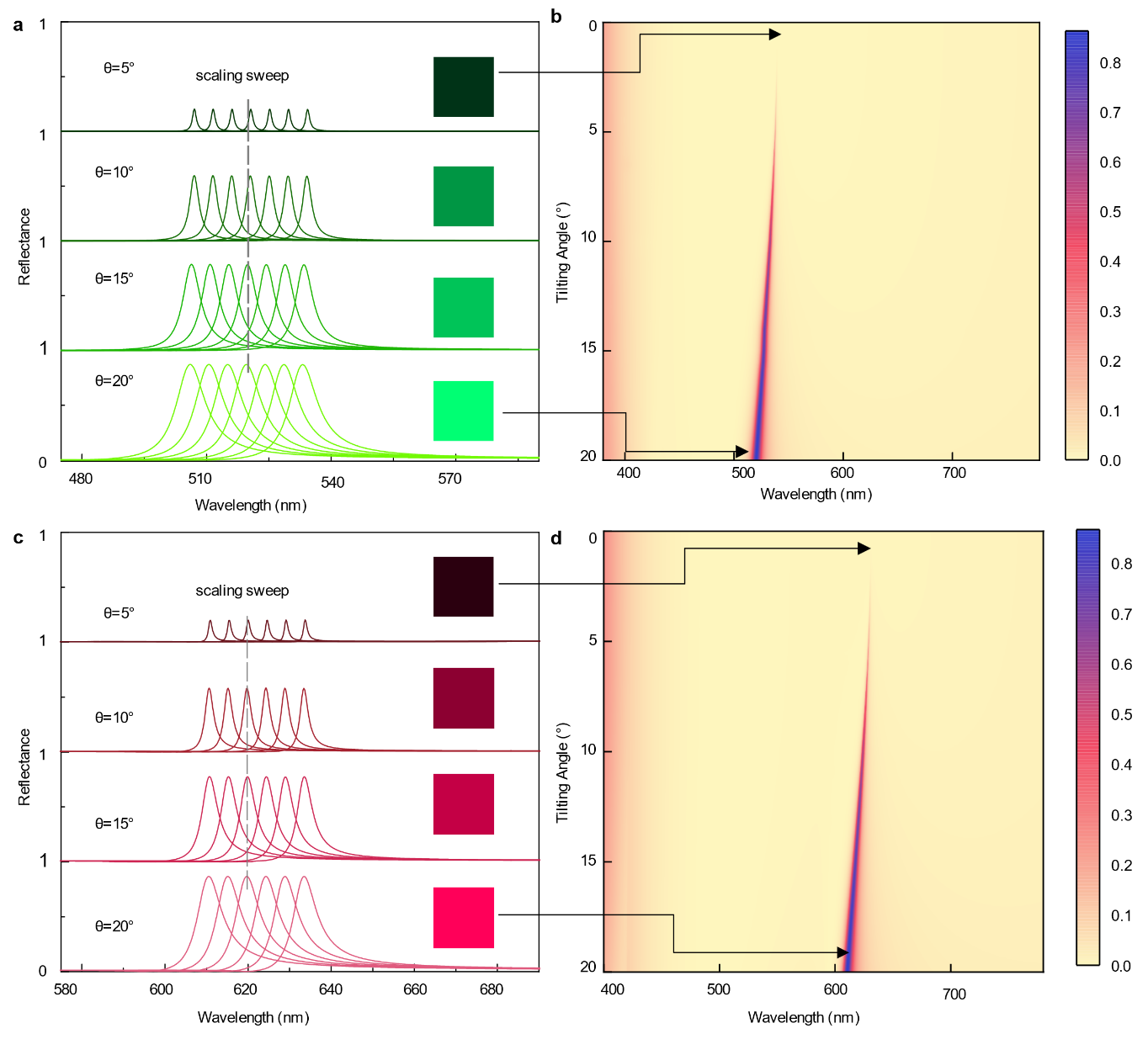


Figure S3. The simulated reflectance spectra of BICs show variations in scaling factors (S) and asymmetries (θ). The scaling factor S is kept fixed at 1.0 (a) and 1.2 (c). The insets display the corresponding calculated colors. The reflectance spectra are analyzed for different tilting angles of the unit cell, while maintaining the scaling factor S fixed at 1 (b) and 1.2 (d).


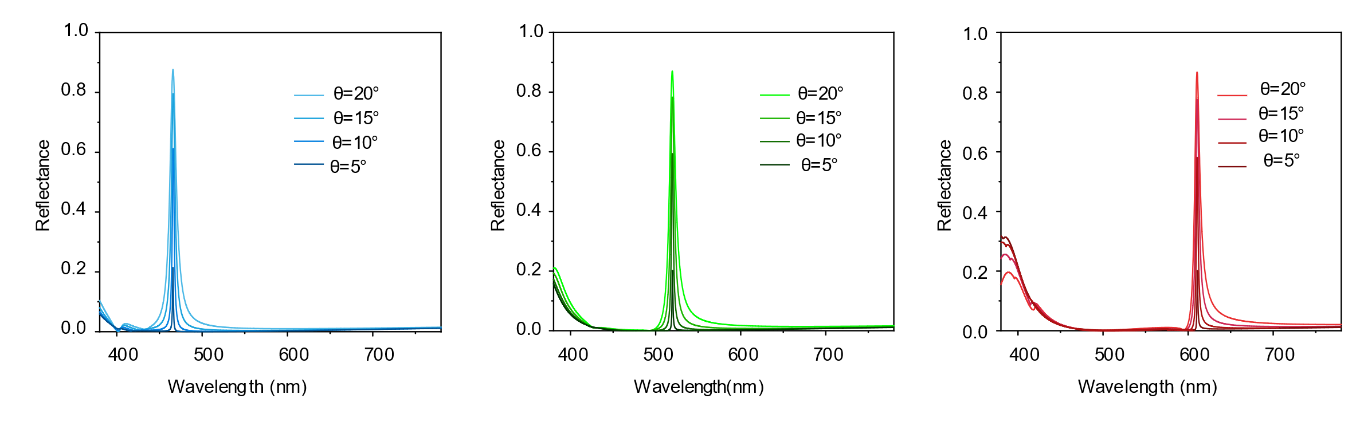


Figure S4. Simulated reflectance spectra of the blue, green and red pixels with the tilting angles of the nanoapertures varying from 0 to 20°.


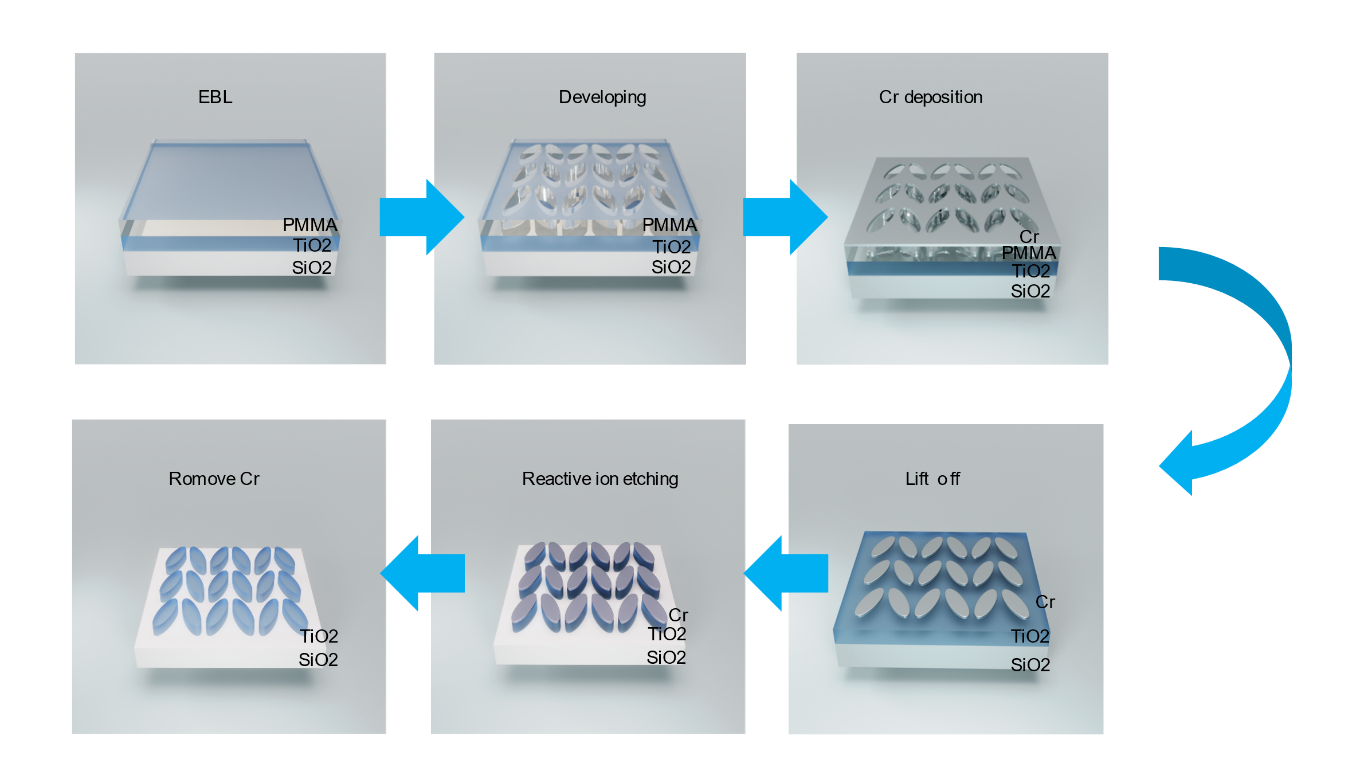


Figure S5. The schematic of the fabrication process for TiO_2_ metasurfaces.

**
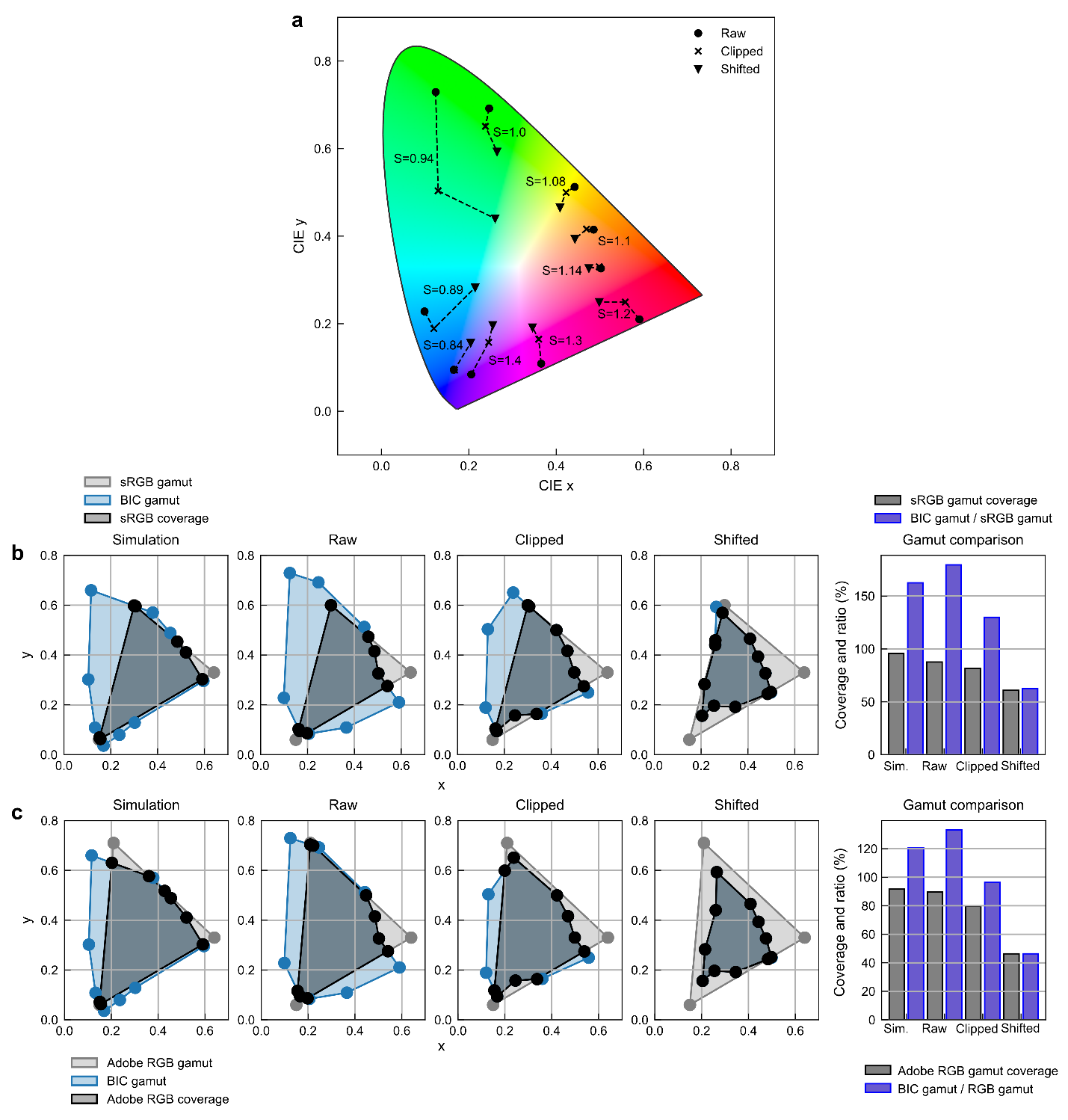
**

Figure S6. Chromaticity coordinates. (a) CIE diagram for unaltered, clipped and shifted experimental spectra. (b) BIC color gamut calculation and comparison with the sRGB gamut and Adobe RGB gamut (c).

**
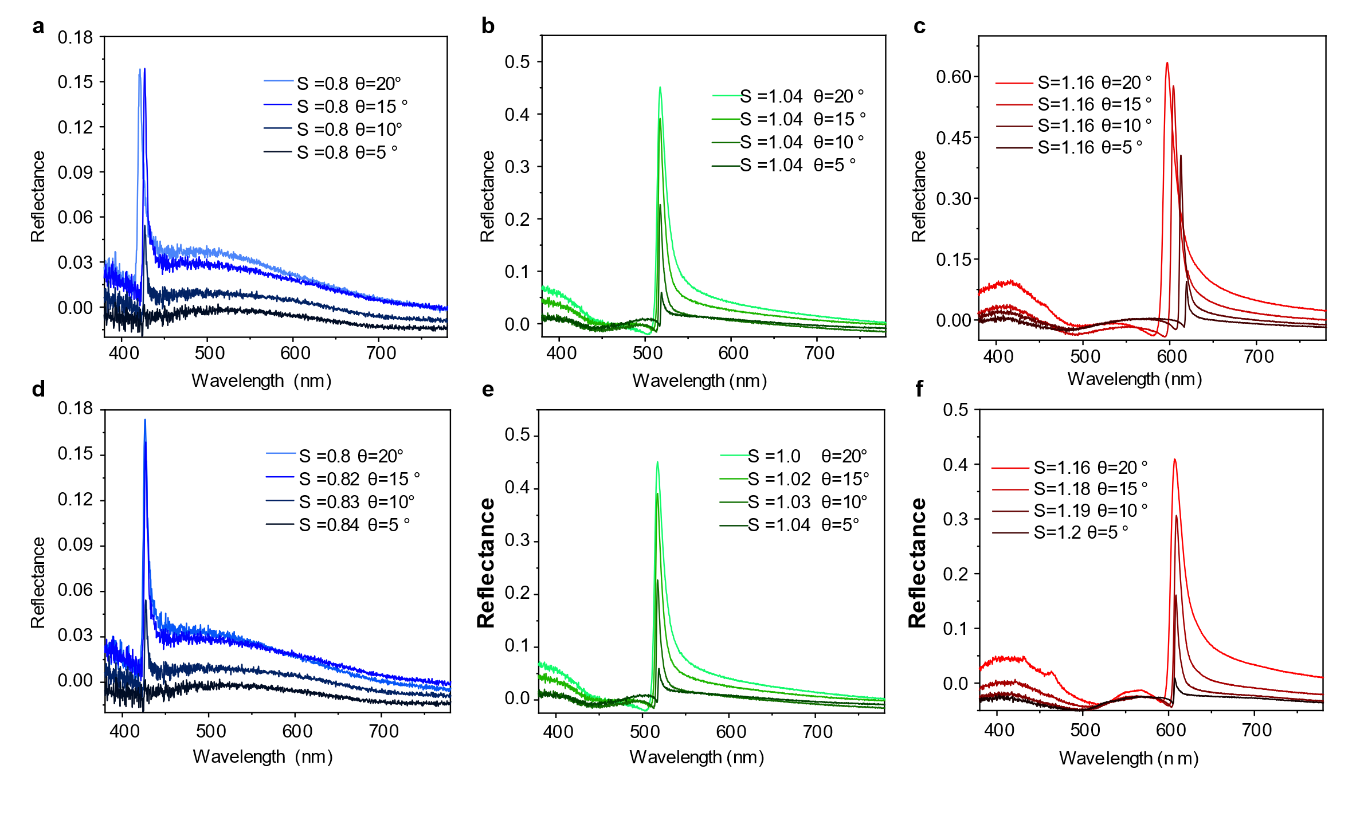
**

Figure S7. Measured spectra of the three primary RGB colors before (a-b) and after(c-f) scaling factor adjustment.

**
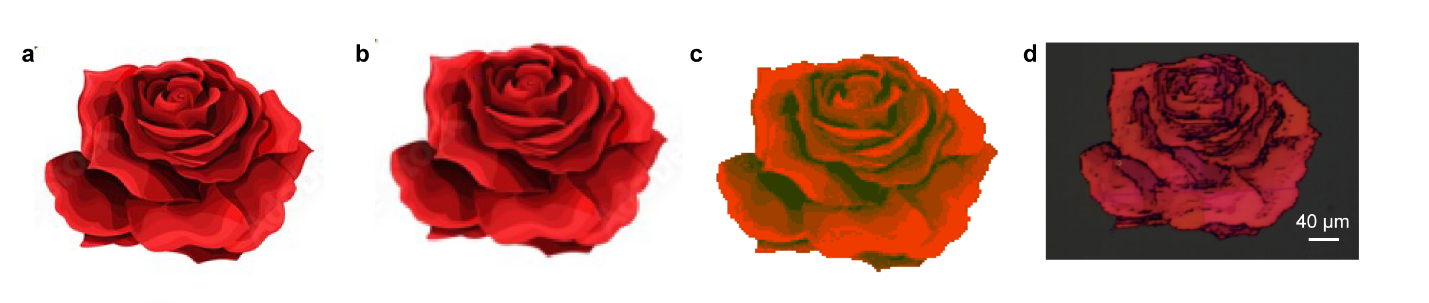
**

Figure S8. A schematic diagram illustrating the design of a patterned metasurface to achieve diverse color hues and brightness simultaneously. (a) A target image. (b) Utilizing the pixelation process to resize the target image to a desired dimension. (c) The pixilated image in settings from the database. (d) Optical experimental micrograph.

**
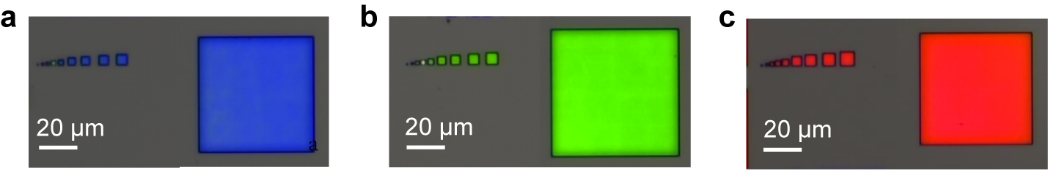
**

Figure S9. Optical images of (a) red, (b)green, and (c)blue TiO_2_ metasurfaces of different areas, the lateral size changes from 60 to 5 μm.


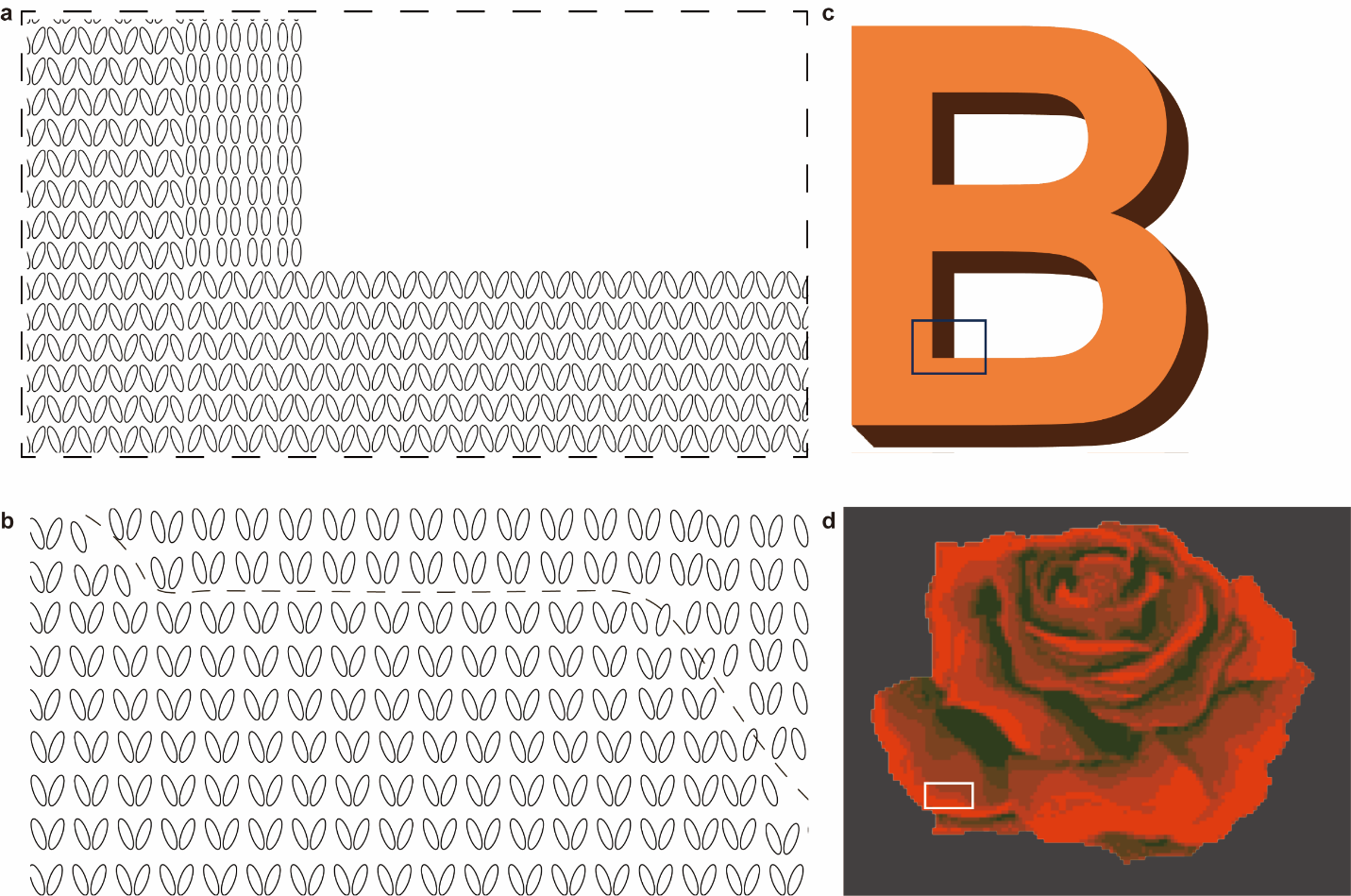


Figure S10. (a-b) CAD images of a selected area showing the array of structures. (c-d) Simulated results of the encoded color image.

References

1. S. Fan, W. Suh, and J. D. Joannopoulos, “Temporal coupled-mode theory for the Fano resonance in optical resonators,” J. Opt. Soc. Am. A 20(3), 569-572 (2003).

2. W. Suh, Z. Wang, and S. Fan, “Temporal coupled-mode theory and the presence of non-orthogonal modes in lossless multimode cavities,” IEEE J. Quantum Electron. 40(10), 1511-1518 (2004).
